# Medical education research: is participation fair?

**DOI:** 10.1007/s40037-014-0120-5

**Published:** 2014-05-21

**Authors:** Kieran Walsh

**Affiliations:** BMJ Learning, BMJ Group, BMA House, Tavistock Square, London, WC1H 9JR UK

## Abstract

Medical researchers and ethicists emphasise the importance of equity, fairness and justice in general medical research participation. No individual or group should be over-represented or under-represented in research—there should be fair participation. Thus far little thought has been given to fair participation in medical education research. There is no evidence based answer as to whether vulnerable groups are ever exploited in medical education research, or whether other individuals or groups are overlooked. However the heavy reliance on undergraduate learners as subjects for medical education research creates two key threats to the fairness of that research. First, there is a risk that undergraduate learners, as a potentially vulnerable population, may be exploited in research settings. Often the faculty carrying out medical education research will be the same faculty that are responsible for delivering medical education and assessing medical students’ competencies. It is possible as a result that medical students might feel pressured to participate in research. Second, there is a risk that other important groups of learners may be inadequately represented. Much medical education research is carried out on undergraduates and relatively little on those who have been doing CPD for many years. Thus much of our research concentrates on only a small proportion of medical learning. The relatively small amount of research carried out on those doing continuing professional development (CPD) is probably because of the difficulties of recruiting and retaining this group of learners in research programmes. Both risks threaten the integrity and usefulness of the resulting research product. Unfair participation in medical education research programmes could have serious repercussions for learners at all levels.

Medical research changed radically in the past century. Then it changed some more. In the first part of the century, too much medical research was carried out on vulnerable people who had not adequately consented to participate. One much cited example is the Tuskegee experiment where African-American men with syphilis went untreated for decades as the researchers sought to study the natural history of the disease [1]. When the story of the Tuskegee experiment came to light in the 1970s, the research establishment moved to protect vulnerable groups and to ensure that they did not bear an undue burden of research participation. With the advent of the HIV epidemic at the end of the century, the opposite problem started to occur. Here a minority group (homosexual men) campaigned for more research into HIV and for their right to participate in that research [2]. Thus the narrative of research ethics changed from moving to protect minorities by ensuring they were not over-represented in trials to moving to protect minorities to ensure they were not under-represented. After just 10 years the pendulum had started to swing back. Today medical researchers and ethicists talk about equity, fairness and justice in research participation [3]. No individual or group should be over-represented or under-represented in research. There is likely still a long way to go to ensure fair participation in medical research—and yet at the same time there is no doubt but that progress has been made.

What can we say about fair participation in medical education research? Are vulnerable groups ever exploited? Are other individuals or groups overlooked or ignored? There is no evidence-based answer to these questions, but certainly it is worth delving into both questions more deeply. Although clinical research ethics and education research ethics are different, important parallels can be drawn between them. They both typically involve experiments where there is potential for harm—albeit sometimes a very small potential. In both types of research there is a power differential between the researcher and the subject of the research. This power differential may be big or small—but it is always there.

With regard to exploitation of vulnerable groups, some would say that undergraduate medical students are a vulnerable group. Medical students may not *appear* to be a vulnerable group because of their apparent sociocultural privilege. Certainly they often come from well-off backgrounds, and are generally able-bodied and well-educated. However, there is no doubt but that medical education research is conducted by people who have a higher position of power than medical students. Often the faculty carrying out medical education research will be the same faculty that are responsible for delivering medical education and assessing medical students’ competencies. Is it possible that medical students as a result might feel pressured to participate in research? Certainly it is possible. Pressure might come from faculty or from peers. It is understandable that some students might agree to participate in education research—perhaps in a desire to ‘please’ their tutors. Worse, in answering research questions, some students might give answers that they hope their tutor-researchers hope to hear. To counteract this, most journals that publish medical education research require researchers to show evidence that they have submitted their research protocol to a research ethics committee and to show evidence that participants have actively consented to take part [4]. This is only as it should be, but a consent form can be a box-ticking exercise and is not always a powerful protection against subtle pressure from seniors. Best practice is to ensure that research team faculty members are blinded to the participation of students they might supervise, requiring recruitment and data collection to be done by a third party and data to be anonymized before the faculty member sees it. This should happen in all medical education research projects at all times and in all places but it is a moot point as to whether this best practice is ubiquitous. Also not all schools have education research committees. All have medical research committees and yet not all of these have the same level of expertise in education research as education research ethics committees have. Many ethics boards are now quite familiar with education research and its potential risks, and require researchers to meet fairly rigorous standards to ensure the safety of participants; however, the gold standard remains approval by an education research ethics committee, which is not always available. Some might argue that medical students might benefit from taking part in medical education research and that is certainly possible. However that does not mean that exploitation has not occurred—some medical students might lose out and this is important even if they are in a minority.

To look at the other side of the coin, is there evidence that all groups have an equal opportunity to actually participate in research? Certainly much medical education research is carried out on undergraduates. A quick read of the main medical educational journals shows that research in undergraduate medical education makes up a sizeable proportion of the publications. Could this be partly because undergraduates are to some degree a captive audience and thus easier to do research on than say fully qualified and autonomous practitioners who have to do continuous professional development (CPD)? These latter learners are heterogenous; have little obligation to participate in research; and can drop out whenever they wish and even when they don’t can be difficult to follow up. Undergraduate medical education lasts four or 5 years, but CPD lasts 30 years [5]. Yet much of our research concentrates on only a small proportion of medical learning.

Is this important? It would not be so important if we could confidently extrapolate what we know about the learning activities of undergraduates to those who have been practising autonomously for 20 years. Yet the activities of the different groups may be quite different and extrapolation a bridge too far. Undergraduate learning is curriculum driven; CPD should be driven by learning needs. Medical students are often highly literate in information technology and will often have spent much of their school years learning in interactive small groups; those who have been doing CPD for many years may be less comfortable in this regard. When viewed in this light, extrapolation seems less likely. And the lack of participation in medical education research of those who need to do CPD seems more troubling.

There could be a problem with regard to fair participation in medical education research. We could be over-investigating the learning habits of undergraduates and at worst unconsciously pressuring them to take part in research. By contrast we may be ignoring more senior doctors who are more advanced in their careers—for a range of different reasons. It is unlikely that there is a conspiracy underlying this or any form of institutional discrimination (as there was with the Tuskegee experiment). It is much more likely that the inconvenience of researching certain groups is deterring investigators. This could have serious repercussions for learners at all levels. This issue should be investigated. Talk of over/under representation suggests that we should give more thought to methodological ideas of sampling to create generalizable findings. It is also important to remember that sampling strategies can be quite different from one research methodology to another. Ensuring representation of vulnerable or minority groups is not always aligned with the principles of sampling rigour for certain research approaches. For the evidence base in medical education to be more sound, we need to explicitly address the tension between what researchers need (or think they need) to conduct valid research and ethical concerns about participation.
